# Piercing the Parliamentary Veil against Judicial Review: The Case against Parliamentary Privilege

**DOI:** 10.1093/ojls/gqac008

**Published:** 2022-05-26

**Authors:** Edward Lui

**Keywords:** constitutional law, constitutional theory, judicial review, parliamentary privilege, separation of powers

## Abstract

For centuries, parliamentary privilege has stood as a bar against judicial review over the internal affairs of Parliament. The literature surrounding parliamentary privilege has mostly been about the scope of the privilege; few have discussed if the existence of the privilege itself is justified. This article undertakes that task, by examining parliamentary privilege as a defence against judicial review. Three propositions will be made. First, in the context of judicial review, parliamentary privilege is defined by the outer limits of the principle of exclusive cognisance. Article 9 of the Bill of Rights 1689 adds nothing. Second, parliamentary privilege as it relates to judicial review is incompatible with the two prevailing models of the separation of powers. Third, six arguments that may be made in favour of parliamentary privilege will be refuted. Accordingly, parliamentary privilege should no longer provide a defence towards judicial review.

## 1. Introduction

It is a long-standing rule that the courts will not interfere with the internal affairs of Parliament. Blackstone famously declared that the whole of the law on Parliament originates from one maxim: ‘that whatever matter arises concerning either house of parliament, ought to be examined, discussed, and adjudged in that house to which it relates, and not elsewhere’.^1^ This echoed what Lord Coleridge CJ said in *Bradlaugh*: ‘What is said or done within the walls of Parliament cannot be inquired into a court of law.’^2^ As we shall see, this highly deferential judicial attitude towards Parliament has been qualified in modern cases. But despite these developments, parliamentary privilege remains a potent defence against judicial review for matters relating to Parliament. It will be contended that parliamentary privilege should no longer provide such a defence. This article will proceed in three parts. First, the two kinds of parliamentary privilege—article 9 of the Bill of Rights 1689 and the principle of exclusive cognisance—will be discussed. It will be contended that, in the context of judicial review, parliamentary privilege is defined by the outer limits of the principle of exclusive cognisance. Second, it will be contended that parliamentary privilege, insofar as it operates as a defence to judicial review, is inconsistent with the separation of powers. Third, six arguments that may be made in favour of parliamentary privilege will be refuted.

This thesis will contribute to the existing literature. The most prominent debates about parliamentary privilege are on *Pepper*^3^ and *Miller (No 2)*.^4^ However, the discussions there were focused on the *scope* of parliamentary privilege; they did not question whether parliamentary privilege may appropriately provide a defence for judicial review against Parliament.^5^ The current thesis hopes to spark a debate on this important, yet largely neglected, question of constitutional law. Before proceeding, two caveats should be noted. First, the discussion below will primarily focus on English law. But as I shall show, parliamentary privilege is by no means English-specific. It exists in many other common law jurisdictions. The main argument made here is also not English-specific: it is based upon the general constitutional theory of the separation of powers. Therefore, the impact of the ensuing analysis may reach beyond English law. Second, parliamentary privilege plays a role in the law beyond judicial review. It also provides a defence against (say) defamation claims^6^ and prosecutions.^7^ This article argues against parliamentary privilege insofar as it relates to judicial review only. It is beyond the scope of this article to discuss how tort law and criminal law operate within Parliament.

## 2. Parliamentary Privilege as a Defence against Judicial Review

In this section, the law on parliamentary privilege as it relates to judicial review will be canvassed. There are two kinds of parliamentary privilege in English law.^8^ The first is based on a statute: specifically, article 9 of the Bill of Rights 1689. It provides that ‘the Freedome of Speech and Debates or Proceedings in Parlyament ought not to be impeached or questioned in any Court or Place out of Parlyament’.^9^ The second is based on common law—commonly known as the principle of exclusive cognisance. It refers to the ‘exclusive right of each House to manage its own affairs without interference from the other or from outside Parliament’.^10^

The relationship between the two kinds of privilege was never precisely demarcated. It is clear they have differences. One is based on primary legislation, the other on common law.^11^ Accordingly, the article 9 privilege may not be waived by Parliament in individual cases: it may only be amended by primary legislation.^12^ In contrast, the common law privilege can be waived.^13^ It may, however, be wrong to treat the two as entirely distinct. In *Prebble*, Lord Browne-Wilkinson described article 9 as ‘merely one manifestation’ of the common law principle.^14^ In a similar fashion, Ekins described the latter as ‘not dependent on the 1689 Act but … evidenced by it’.^15^ In *Chaytor*, Lord Rodger even suggested that unless a matter falls within the principle of exclusive cognisance, article 9 cannot possibly apply.^16^ There is thus only ‘one basic question’: is any matter one ‘within the exclusive jurisdiction or cognisance of Parliament’? If not, article 9 does not offer any independent protection.^17^ It must be noted that in *Chaytor*, Lord Phillips did not apparently go so far as to subsume article 9 within the principle of exclusive cognisance entirely:^18^ but, somehow oddly, the other judges agreed with both Lord Phillips and Lord Rodger’s judgments.^19^ In any event, it suffices to show that the two kinds of privilege are—whether one accepts Lord Rodger’s position or not—highly related and intertwined.

What, then, is the scope of the two kinds of privilege? No effort will be made to comprehensively summarise all the technical niceties on this topic.^20^ However, the scope of the privilege as it relates to judicial review will be canvassed. I will turn first to article 9. Proceedings in Parliament has been defined to include ‘some formal action, usually a decision, taken by the House in its collective capacity’. This extends to the ‘whole process … by which it reaches a decision’—such as the process of debate.^21^ Despite this apparently broad formulation, Lord Phillips in *Chaytor* added an important qualification. He held that the article principally relates to ‘freedom of speech and debate’ in Parliament—being ‘the core or essential business of Parliament’.^22^ Also, article 9 should be given a ‘narrow ambit that restricts it to the important purpose for which it was enacted’—being the ‘freedom of Parliament to conduct its legislative and deliberate business without interference from the Crown or the Crown’s judges’.^23^ If a matter does not relate to ‘the core or essential business of Parliament’—such as an MP’s submission of claim forms for allowances—it will not be covered by the privilege.^24^

I now turn to the principle of exclusive cognisance. As mentioned above, this refers to the ‘exclusive right of each House to manage its own affairs without interference from the other or from outside Parliament’.^25^ In *Chaytor*, Lord Phillips cited with approval a report by Parliament, which noted that a reference to the internal affairs of the House

are *loose* and *potentially extremely wide in their scope*. On one interpretation they embrace, at one edge of the spectrum, the arrangement of parliamentary business and also, at the other extreme, the provision of basic supplies and services such as stationery and cleaning.^26^

The report then provided a definition of the common law privilege: it only covers areas which are ‘so closely and directly connected with proceedings in Parliament that intervention by the courts would be inconsistent with Parliament’s sovereignty as a legislative and deliberate assembly’. This would include, for instance, the Speaker’s decision on the ‘facilities within the precincts of the House’.^27^*Miller (No 2)* shed some further light on this point. The Supreme Court held that since prorogation does not relate to ‘the core or essential business of Parliament’—ie the freedom of speech and debate in Parliament—neither article 9 nor the principle of exclusive cognisance applied.^28^ Subject to the further analysis below, this may suggest that the principle of exclusive cognisance has a similar scope to that defined in the report.

The two principles together culminate in a position whereby a judicial review of parliamentary matters will be shielded. Three authorities illustrate this. In *Bradlaugh*, the plaintiff was elected to the House of Commons. The Speaker refused to let him take his oath. The House also resolved to exclude him from the House. The plaintiff applied to the court for an order that he shall not be so removed. He contended that the resolution of the House was ‘illegal’ and ‘deprived both him and the electors … of a right recognised by the law, which ought to be protected by the law’.^29^ The court refused to grant the order sought. As mentioned at the start of this article, Lord Coleridge CJ held that ‘What is said or done within the walls of Parliament cannot be inquired into [in] a court of law’.^30^ The jurisdiction of the Houses over their own members is ‘absolute and exclusive’.^31^ Similarly, Stephen J held that ‘the House of Commons is not subject to the control of Her Majesty’s courts in its administration of that part of the statute law which has relation to its own internal proceedings’.^32^ In *McGuinness*,^33^ the applicant was a member of Sinn Féin. He was elected to Parliament, but he refused to take an oath. The speaker decided that he should not be allowed to use the facilities in the precincts of Parliament. He applied for judicial review of the Speaker’s decision. Kerr J held that, quite regardless of whether it fell within the scope of article 9, ‘the Speaker’s action lies squarely within the realm of internal arrangements of the House’. It was thus ‘not amenable to judicial review’.^34^

In these two authorities, it was not made clear how the scope of article 9 and the principle of exclusive cognisance differed (if at all). In particular, it was not made clear whether article 9 might cover any grounds beyond the principle of exclusive cognisance. But we can attempt to supplant this by way of analysis. In both the leading cases of *Prebble* and *Chaytor*, article 9 has been described as the narrower principle.^35^ This proposition has been adopted in other cases, such as *Fayed*^36^ and *McGuinness*. While *Miller (No 2)* seems to suggest that they have a similar scope—ie that they only relate to ‘the core or essential business of Parliament’^37^—the court never confronted the remarks in *Prebble* and *Chaytor* directly. Rather, the court cited *Chaytor* with approval.^38^ It is doubtful if the court in *Miller (No 2)* was going so far as to equate the scope of article 9 with the principle of exclusive cognisance entirely, regardless of context. The more likely possibility is that the court was simply saying, in the context of prorogation, that neither privilege is wide enough to avail the UK government.

I will now return to *Chaytor*. If I adopt Lord Rodger’s view, the matter will be made simple: article 9 adds nothing to—and is simply subsumed within—the principle of exclusive cognisance. What, then, of Lord Phillips’s view? While he did refer to *Bradlaugh* in his discussion of article 9, he made clear that the decision does ‘not distinguish between the narrow privilege under article 9 and the broader exclusive cognisance of Parliament’.^39^ He made no further reference to judicial review when discussing article 9. This suggested that article 9 brings little to the table when it comes to judicial review. By contrast, in discussing the principle of exclusive cognisance, he specifically mentioned that in judicial review ‘The courts will respect the right of each House to reach its own decision in relation to the conduct of its affairs’.^40^ He cited *McGuinness* and *Fayed* as illustrations of how the principle of exclusive cognisance applied to ‘claims for judicial review in relation to conduct by each House of its internal affairs’.^41^ These cases were not, however, cited in his discussion of article 9. The stark contrast showed that he categorised these cases—where judicial review was barred by parliamentary privilege—as coming under the principle of exclusive cognisance. All these points support the suggestion that Lord Phillips, in the context of judicial review, agreed with Lord Rodger: article 9 offers no additional defence to judicial review than the extent to which the principle of exclusive cognisance does. This is also consistent with Lord Phillips’s views that article 9 should be given a ‘narrow ambit’^42^ and that article 9 was the narrower principle between the two.^43^

This analysis finds additional support in the third case, *Leung*.^44^ Some members of the Legislative Council in Hong Kong attempted to filibuster the Council by proposing numerous amendments in the debate. The president of the Legislative Council ruled that the debate should end and a vote be cast. One such member applied for judicial review, arguing that the decision by the president was *ultra vires*, as the internal rules of the Legislative Council did not authorise him to close the debate. It was held that, deriving from the doctrine of separation of powers, ‘the courts will recognise the exclusive authority of the legislature in managing its own internal processes in the conduct of its business’. Accordingly, ‘the courts will not intervene to rule on the regularity or irregularity of the internal process of the legislature but will leave it to determine exclusively for itself’.^45^ As a result, the court held that as long as the president had such a power to end the debate, ‘It is not for this Court to consider whether or not this power was properly exercised’.^46^ Two points are noteworthy from this decision. First, the principle propounded in *Leung* is highly similar to the principle of exclusive cognisance in English law.^47^ Both require courts to refrain from interfering with the internal affairs of the legislature, based on the need to recognise its exclusive authority over such matters. Accordingly, the principle that the court will not rule on the regularity of the processes—such as on the Speaker’s exercise of his powers—will likely also apply in English law. Indeed, one may say that *Bradlaugh* and *McGuinness* are themselves illustrations of this principle. Second, in reaching its conclusion, the court did *not* refer to the Hong Kong counterpart of article 9, ie section 3 of the Legislative Council (Powers and Privileges) Ordinance (Cap 382).^48^ Thus, it supports the foregoing analysis that article 9 adds nothing to the principle of exclusive cognisance in providing a defence to judicial review.

The above-mentioned analysis does not only canvass the law of parliamentary privilege; it also defines the target of attack for this article. Without this section, it may be asked: when it is suggested that parliamentary privilege violates the separation of powers, what does parliamentary privilege mean? This section shows that, in the context of judicial review, the principle of exclusive cognisance represents the entirety of what parliamentary privilege offers as a defence to judicial review. Except for a difference in waiver (which is not here material), article 9 adds nothing. Therefore, parliamentary privilege in the subsequent analysis refers to the outer limits of the principle of exclusive cognisance. Article 9 is, in this respect, nugatory.

## 3. The Separation of Powers and Parliamentary Privilege

In this section, it will be contended that parliamentary privilege—insofar as it offers a defence to judicial review—is incompatible with the separation of powers. This is true for both of the prevailing models—the liberty model and the efficiency model. Accordingly, parliamentary privilege, in the context of judicial review, is normatively unjustified.

### A. The Separation of Powers in the UK

A preliminary question is whether the UK has a separation of powers altogether. This point can be easily dismissed, but an effort will be made to establish this fundamental point. Barber has argued that in whatever state, there will have to be a separation of powers.^49^ In order for a state to effectively ‘control’ its ‘territory and people’, it needs an executive (eg a law enforcement agency).^50^ It also requires the courts to ‘create’, ‘apply’ and ‘adjudicate on’ legal matters, since the state will have to rule through law.^51^ The legislature is needed to enable ‘a democratic government’ within the state.^52^ No one would deny that the UK has such needs. The simple point is therefore established: the UK requires these institutions and, to some extent, the separation of powers.

The above only establishes that these separate institutions must exist within the UK. It does not show the *extent* to which they are separated. For instance, Barendt has suggested that ‘there is no effective separation of powers between the executive and the legislature in the United Kingdom’. This is because, unless there is a significant party split, the government will effectively control the legislature through its majority in Parliament. And the majority—coming from the same party—will naturally be inclined to support the government.^53^ Two points can be made against this. First, Barendt’s view has been doubted. Barber has argued that there is a ‘deep divide’ between the executive and legislative branches in the UK. Only the minority of the executive—being the political members—sit in Parliament.^54^ Barendt’s argument is limited to this small extent of overlap. It cannot apply to the majority of the executive—such as civil servants, soldiers and NHS workers. For instance, the recruitment of civil servants is largely ‘[insulated]... from political interference’ that may arise from the ministers and the prime minister.^55^ It is perforce false to say that the executive and legislative branches of the UK are generally not effectively separated. Second, in any event, Barendt accepts that ‘there is... an effective separation of judicial power from the other branches’.^56^ Therefore, Barendt’s argument does not preclude the current thesis—which is premised upon the separation of powers between the judicial and legislative branches. Yet, despite these points, Barendt’s argument has force: it shows that there is relatively little separation between Parliament and the ministerial level of the UK executive.

### B. The Liberty Model

One of the main models of the separation of powers is the liberty model. According to this model, the primary purpose of the separation of powers is to avoid abuses of power by the government.^57^ The model assumes that ‘every political actor will seek to maximise his own political influence’.^58^ By separating the governmental powers to different political actors, it creates ‘friction’ between them. It will be more difficult for one political actor to abuse his power^59^—since to do so he may require the consent of another actor (eg the executive attempting to pass a bill in Parliament) or may be met with checks from another actor (eg the judicial review of executive action). This, in turn, prevents tyranny and protects the liberty of those being governed.^60^

Therefore, according to this view, it is objectionable that a certain political actor has a conclusive, unquestionable power—for the simple reason that such a power will be prone to abuse. It contravenes the requirement that governmental powers must be divided and shared amongst multiple actors—so that the ‘concentration of powers’ can be avoided.^61^ This thinking has long influenced English judicial thought. A prominent example is the law on ouster clauses in English administrative law. The courts have strenuously resisted the exclusion of judicial review by virtue of these clauses.^62^ This is to ensure that the decision maker may not have a conclusive say when interpreting its power-conferring statute: its interpretation must be subject to judicial scrutiny.^63^ Otherwise, it will confer on the decision maker an ‘unlimited power to determine such limit [of its own power] at its own will and pleasure’—a power aptly described as ‘autocratic’.^64^

The argument which follows is a simple one. By upholding parliamentary privilege as a defence of judicial review, it grants an exclusive authority of Parliament over matters that fall within the privilege. This power will be entirely unchecked by the other branches. It will not be checked by the executive, for two reasons. First, it will be contingent upon Parliament deciding to grant it statutory power to interfere with Parliament itself (in which case it is doubtful if it is a check by the executive or a check by Parliament). Even if such a check is initially imposed, it will be removable at the whim of Parliament. Second, as Barendt explained, there is little ‘effective separation of powers’—and consequently ‘checks and balances’—between the ministerial level of the UK government and Parliament.^65^ Since the ruling party effectively controls Parliament, it will have little incentive to move for checks over the internal affairs of Parliament. The rest of the executive (eg the NHS workers) will not have a say in this, although they are effectively separated from Parliament.^66^ This power will also not be checked by the judiciary, because parliamentary privilege will preclude judicial review. *Bradlaugh* and *McGuinness* exemplify this. The result is that Parliament will have a conclusive, unquestionable power over matters within parliamentary privilege. This power will be entirely unchecked, and thus contravenes the liberty model of the separation of powers.

There may be two objections to this. The first objection is that Parliament is already democratically accountable, regardless of judicial review. If its members (whether it be the Speaker or the government’s majority in Parliament) decide to abuse its powers, there will surely be political repercussions. Accordingly, it is wrong to say that parliamentary privilege gives Parliament an unchecked power. There are checks: they are just in the form of political pressure, not legal constraints. This objection fails. First, the thesis now is that parliamentary privilege contravenes the separation of powers, because there are no checks and balances from the other branches. Apart from Parliament, there are only two other branches in the liberty model: the UK government and the judiciary. This objection does not rely on these branches. Rather, it relies on the voters—something external to the separation of powers. This is simply inadequate to respond to the present critique that the theoretical model has been infringed. To complete his argument, the critic must show that the voters should be regarded as a fourth branch of the separation of powers and so, through the voters, the liberty model is nevertheless satisfied. Unless the critic proves this proposition, his objection cannot succeed. Second, in any event, this view assumes that this fourth branch provides some effective checks and balances: so, if there is an abuse of power shielded by parliamentary privilege, there will be political repercussions. It is theoretically possible that some branches of government will not provide effective checks and balances towards each other: this is why Barendt could make his above-mentioned argument about the UK government and Parliament. It is contestable if the voters do provide such checks. Voters are not all educated constitutional lawyers; they may not necessarily react strongly to abuses of power. Their votes may also be swayed by other factors (eg a voter may prefer a party—despite a dubious track record on abuses of power—because of its stance on a controversial political issue).

The second objection is that there is a limit to parliamentary privilege. Despite the principle, there is still a limited degree of judicial scrutiny. It is now settled that it is for the court—not Parliament—to determine ‘the scope of [p]arliamentary privilege’.^67^ So at least, per this objection, the court will be able to determine the outer limits of the privilege. This objection is weak. A critic making this argument would agree that there is a need to avoid abuses of power by distributing governmental powers: his suggestion is that the court’s role in determining the scope of the privilege fulfils this need. But this does not address the heart of the concern: that some undoubtedly privileged powers (eg the Speaker’s power to regulate Parliament) may be abused. It does not matter whether the court gets to have a say on the scope of the privilege. The courts, after all, must apply the established legal principles: they cannot directly say ‘since you have abused your privilege, its scope will now be curtailed’. The only way in law to curtail the scope of parliamentary privilege is if Parliament waives or relinquishes its own privilege. The courts may not compel the Parliament to waive its privilege.^68^ Indeed, in *Chaytor*, Lord Phillips noted that ‘extensive inroads have been made into areas that previously fell within the exclusive cognisance of Parliament’.^69^ But this was not an effort by the courts: it was an effort by Parliament to make ‘[s]tatutory inroads’ into its own privilege.^70^

### C. The Efficiency Model

The second model of the separation of powers is the efficiency model. Unlike the liberty model, it does not act primarily to restrict governmental action so as to prevent governmental power from being abused. Rather, it aims to ‘facilitate’ governmental power and promote efficient ‘state action’.^71^ It does so by allocating governmental powers to state actors which have the most ‘suitable’ composition, ‘expertise’ and ‘decision-making process’.^72^ In this regard, Barber has made an impressive account as to the different features of the executive, legislature and judiciary: and as to why they are allocated the functions that we usually see they are allocated today. The legislature is ‘large’, so that it may incorporate a considerable range of opinions in its decision-making process. A majority of its members do not have ‘specialist knowledge’ and those members are, in Barber’s words, ‘amateurs’. This is because the members are not chosen due to their expertise; rather, they are chosen for their abilities to represent their constituents.^73^ In comparison, the executive ‘possess[es] technical capacities that the legislature lacked’. They ‘are often appointed because of their technical knowledge of the area they work in’—such as expertise in ‘foreign affairs’ and in ‘military’ matters.^74^ The judiciary possess legal expertise, but they have little expertise in policy and limited information (being ‘limited to that supplied by the [litigants]’): they are therefore unequipped to make political decisions.^75^ This explains the current division of governmental power in the UK. The legislature, with its general lack of expertise, is unable to form detailed public policy. The executive will do that with its expertise. But the policy must be understood and approved by the ‘amateur’ legislature: by doing so, the legislature forces the executive to be accountable to the ordinary people that lack such expertise.^76^ The courts will not be involved in this process: their role is limited to adjudicating when questions of law arise. Kavanagh calls this a ‘joint enterprise of governing’, whereby ‘each branch...contributes different elements’ that ‘reflect their particular institutional structures’ and ‘skills’.^77^

It is submitted that parliamentary privilege is incompatible with this model. Parliamentary privilege covers many matters which are otherwise perfectly justiciable questions of law. A key example is *Leung*.^78^ According to this, the irregularity of an internal decision within Parliament will not be judged by the courts; rather, it will be judged exclusively by Parliament itself. But whether a decision is irregular involves a question of law. For instance, a decision may be made with illegality (ie a rule of parliamentary procedure has been misinterpreted), with procedural unfairness or with irrationality. Imagine a case like *Bradlaugh*, but with slightly modified facts: for example, say that Parliament resolved to evict all the Green Party MPs for their purportedly over-aggressive environmental policies. Those MPs were not given any chance to explain themselves before the vote (procedural unfairness) and were evicted for a matter that entirely does not justify it (irrationality). This may also involve issues of human rights: indeed, the applicant in *McGuinness* later took his case to Strasbourg, alleging a violation of his freedom of expression.^79^ Are these questions of law that MPs—including the Speaker—can properly decide? At best, the Speaker who is an expert with *Erskine May*^80^ may know the rules of parliamentary procedure perfectly well, and may adjudicate on matters of illegality (even so, it is contestable if he can measure up to a professional judge). But the other matters, it is suggested, involve thick layers of technicalities and are more appropriately judged by the courts. This resonates with Barber’s account above. The MPs are not elected by reason of their expertise: they are mostly amateurs. Their presence is justified to represent the broad range of electorates. Judges, on the other hand, are experts in matters of law. Questions of administrative law and human rights—whether they take place within or outside Parliament—should be most appropriately judged by the courts. This has nothing to do with democratic representation, a matter for which the Parliament is more suitable. Parliamentary privilege—which places these questions of law within the exclusive realm of Parliament—is therefore contrary to the efficiency model.

There are two possible objections to this proposition. First, it may be suggested that these internal affairs of Parliament are frequently highly political. They therefore consist of matters of politics, not matters of law. Accordingly, per Barber’s argument, such matters are more suited for the politically constituted Parliament than for judges.^81^ There is no question that matters within Parliament frequently occur in a political context. Indeed, important questions of law frequently arise in such contexts (one need look no further than the two *Miller* litigations). An important distinction must, however, be drawn between political questions and legal questions that arise in a political context. In *Miller (No 2)*, the court explained:

although the courts cannot decide political questions, the fact that a legal dispute … arises from a matter of political controversy, has never been sufficient reason for the courts to refuse to consider it … almost all important decisions made by the executive have a political hue to them. Nevertheless, the courts have exercised a supervisory jurisdiction over the decisions of the executive for centuries.^82^

If one challenges the Speaker’s decision as being tainted with illegality, procedural impropriety or irrationality, the challenge raises questions of law—albeit ones housed in a political context. The adjudicator will have to determine, respectively, whether there has been a misinterpretation of the rules of parliamentary procedures,^83^ whether the criterion of fairness has been breached^84^ and whether the *Wednesbury* test has been satisfied.^85^ These require the application of legal standards—and thus are more suited for judges to decide. This is very different from a political challenge to the Speaker’s decision, which is divorced from the legal standards. It is fairly widely accepted that such a matter is more appropriately determined by Parliament. But these are matters which did not concern us to start with. We are solely concerned with what parliamentary privilege offers as a *legal* defence.

The critic may then slightly alter his argument. He may argue that decisions within Parliament could be administrative, rather than political: and matters of internal administration should be dealt with by Parliament, not courts. He would cite *McGuinness* as a case about the administration of facilities within Parliament. This argument similarly fails, for two reasons. First, it is difficult to disentangle administrative decisions from political ones. While *McGuinness* can be described as an administrative decision about facilities, it undoubtedly has a key political theme to it: about whether the members of Sinn Féin, despite their refusal to take the parliamentary oath, should be excluded from Parliament. It is difficult to draw a legal distinction on such lines: that if the decision is political, parliamentary privilege applies, but if the decision is administrative, the privilege does not. Second, it remains that administrative decisions within Parliament also only raise questions of law, as far as the courts are concerned. The courts are not concerned with whether these administrative decisions were rightly made—unless they engage the grounds of judicial review (eg rationality review). Indeed, this is how the courts have long reviewed similar decisions made by the UK government.^86^

## 4. Rebuttals: Arguments for Parliamentary Privilege

So far, we have seen that parliamentary privilege is incompatible with both prevailing accounts of the separation of powers—the liberty and efficiency models. Some objections which relate to the models have already been addressed. But there are still some arguments that may be made in favour of parliamentary privilege. Six of them will now be addressed separately. It will be contended that none of them suffices to salvage parliamentary privilege as a defence to judicial review. It should therefore be discarded for being inconsistent with the separation of powers.

### A. The ‘Pure Doctrine’ and the Province of Parliament

The critic may first rely on the ‘“pure doctrine” of the separation of powers’. This is known as the classic formulation of the separation of powers, famously promulgated by Vile.^87^ The doctrine provides that the government should be divided into the three branches of government: the legislature, the executive, and the judiciary’. Then each branch will have an ‘identifiable function’. It will be, respectively, that of enacting laws, of executing them and of adjudicating cases. Each branch of government must be strictly ‘confined to...its own function’, and it must not ‘encroach upon the functions of the other branches’.^88^ The critic will then say that, given this view, the courts should not encroach upon the province of Parliament. Parliamentary privilege precisely prevents the courts from interfering with Parliament’s performance of its legislative functions. This has been echoed by the authorities on parliamentary privilege. In *Bahamas*, Lord Nicholls explained that parliamentary privilege

is essential to the smooth working of a democratic society *which espouses the separation of powers between a legislative Parliament, an executive government and an independent judiciary*. The courts must ever be sensitive to the need to *refrain from trespassing, or even appearing to trespass, upon the province of the legislators*.^89^

The pure doctrine also underlies the court’s reasoning in *Leung*. There, the court emphasised that the separation of powers in Hong Kong required the court to ‘recognise the *exclusive* authority of the legislature in managing its own internal process in the conduct of its business, in particular its legislative processes’.^90^ From this, the court inferred that it will ‘leave [the legislature] to determine *exclusively* for itself’ the regularity of its internal processes.^91^

Two points may be made in response. First, the pure doctrine is highly impracticable. It is doubtful if any modern state is conforming to this doctrine. Many, if not all, state actors perform more than one governmental function.^92^ Apart from enforcing laws, the executive also enacts delegated legislation and plays a key role in the legislative process (eg drafting legislative bills).^93^ Apart from adjudicating, ‘the courts also make...law...within certain limits’.^94^ Therefore, the premise of the objection—that the pure doctrine must be adhered to—is highly questionable.

Second, even if we apply the pure doctrine, it is unclear if parliamentary privilege is justified. Parliament’s role under the doctrine is to enact laws. In comparison, the court’s role is to adjudicate. If we apply this stringent division, and insist that neither body should trespass into each other’s role, the natural conclusion is that the *adjudication* of questions of law within Parliament should be performed by courts. Indeed, the pure doctrine would further strengthen this position. It would require further that Parliament should not interfere with the court’s adjudicative function; and it should focus on enacting laws *only*. Therefore, the critic’s interpretation of the pure doctrine (eg that in *Bahamas*) is doubtful.

### B. Self-defence Mechanism

The critic may then rely on Barber’s thesis on ‘self-defence mechanisms’. According to Barber, there are two kinds of self-defence mechanisms in a constitutional order: negative and positive self-defence mechanisms.^95^ We are concerned here with the former kind. Negative self-defence mechanisms seek to ‘protect’ one body ‘from the unwarranted attention of another body’. Parliamentary privilege is itself an example: it ensures that the internal affairs of Parliament will not be subject to judicial review.^96^ Barber has explained that self-defence mechanisms possess an important feature: they flout the efficiency account. They do not exist to allocate powers to the most suitable state actor. Rather, they prevent a body ‘from exercising a power that appears suitable for it to exercise’.^97^ In the case of parliamentary privilege, the critic will argue that while it is most suitable for the judiciary to adjudicate on questions of law that arise in Parliament, the privilege exists to curtail this judicial function and therefore protect Parliament.

The critic’s argument, however, cannot stop here. As Barber explained, a self-defence mechanism is necessary in a constitutional order only to perform two functions. Normally, state actors interact in ‘comity’: they support each other towards the same aim. But self-defence mechanisms set them in ‘friction’: it causes the institutions to ‘[s]train against each other’.^98^ By doing so, a self-defence mechanism performs two functions. First, it militates against mistakes by state actors. State actors may be particularly vulnerable to certain kinds of matters: for instance, Parliament may be ‘at risk of the vices of majoritarianism’, and courts may be ‘tempted to make decisions on matters’ they have little knowledge of.^99^ Second, it ‘[divides] constitutional labour’ and establishes an ‘invisible hand system’. By the latter, it refers to a system whereby different state actors are assigned to ‘consider that aspect of the issue it is ‘best placed to assess’—and their efforts will be combined by the constitution into ‘a collective decision that pursues the common good as a whole’.^100^ ‘The division of constitutional labour’ is to ‘[ensure] that institutions play to their strengths’, so that they may undertake ‘those tasks they are best suited to undertake’.^101^ For instance, Parliament is ‘good at gauging the public interest’. The courts are ‘good at recognising the value of rights’. By creating friction between Parliament and the courts, an invisible hand system is established. Their conflict could lead to ‘the totality of common good’—the laws will cater for both public interest and rights.^102^ Per the foregoing analysis, a self-defence mechanism is *prima facie* unjustified for flouting the efficiency model of the separation of powers. It is *only* justified in the constitutional order when it fulfils either or both of these purposes: and the critic must show that parliamentary privilege satisfies this to sustain his argument.

First, parliamentary privilege does not guard against mistakes. It does not guard against mistakes made within Parliament itself because it protects such decisions from judicial review. Per *Leung*, the court will not examine if a power has been properly exercised within Parliament. Even a reckless approach towards the propriety of decisions made within Parliament will be shielded. It may then be suggested that, nevertheless, parliamentary privilege helps guard against mistakes made by the courts—by preventing them from stepping onto ground they know little about (ie the internal affairs of Parliament). This view ignores the fact that in judicial review, the courts are only concerned with questions of law. It cannot be plausibly suggested that courts know little about how to conduct statutory interpretation, procedural review and rationality review. The critic may then respond by saying that while this is true, these questions are now applied in a parliamentary context. But as *Miller (No 2)* makes clear, the courts are familiar with conducting judicial review that exists in a political context. Nor does it matter that this political context happens to be parliamentary.^103^ Further, if the review concerns empirical and factual questions about parliamentary history and practice, the courts have demonstrated their capability to have a good grasp of them—one need look no further than *Chaytor* and the impressive historical survey conducted by Lord Phillips, when he sought to determine whether Parliament had waived its privilege over criminal cases.

Second, parliamentary privilege does not establish a successful invisible hand system. The premise of the invisible hand system is that each institution is assigned to ‘consider that aspect of the issue it is best placed to assess’. It ensures that the institutions ‘play to their strengths’.^104^ This is not what parliamentary privilege does. As explained above, it relies on Parliament—not courts—to deal with questions of law that arise inside Parliament. Further, the invisible hand system set up by parliamentary privilege makes little sense. As Barber suggested, in an invisible hand system involving Parliament and the courts, Parliament would be responsible for recognising the public interest and the courts would be responsible for ‘recognising the value of rights’.^105^ One may ask rhetorically: how do the facts of *Bradlaugh* and *McGuinness* (exclusion of MPs) and *Leung* (irregularity in procedures) concern any questions of public interest? Of course, if one defines ‘public interest’ very broadly, almost every judicial review can be about the public interest. But we are now discussing questions of public interest *for which Parliament is better placed* than the courts to assess. They could be matters involving social values (eg abortion or same-sex marriage) or socio-economic policies (eg national budgets). The subject matter of these cases is nothing similar to those concerned by parliamentary privilege. Rather, they concern questions of rights. It may be immediately objected that in *Bradlaugh*, *McGuinness* and *Leung* we were not concerned with convention rights. This can be accepted. But even so, the decision will engage important questions of *interests* for the aggrieved MPs. As Craig argued, interests and rights cannot be easily segregated: and should not be treated as if they were radically different.^106^ This all shows that if an invisible hand system is to be set up on the internal affairs of Parliament, it should be the courts—which are best suited to recognise the question of rights—that play that role.

### C. The Need for Comity

The critic may then resort to Joseph’s thesis: that parliamentary privilege ‘preserve[s] the political–judicial balance’ in the UK constitution. Joseph referenced parliamentary history: when the courts have established that it may decide ‘the existence and scope of’ parliamentary privilege, Parliament has ‘never formally acquiesced in [this] ruling’; it has insisted that it is the ‘sole judge of its [privilege]’. Therefore, the courts have long been aware of a ‘potential for collision’ between the two branches if the judiciary curtails parliamentary privilege.^107^ In *Pickin*, Lord Simon explained:

It is well known that in the past there have been dangerous strains between the law courts and Parliament—dangerous because each institution has its own particular role to play in our constitution, and because collision between the two institutions is likely to impair their power to vouchsafe those constitutional rights for which citizens depend on them. So for many years Parliament and the courts have each been astute to respect the sphere of action and the privilege of the other …^108^

Therefore, this ‘potential for collision’—which in turn may lead to a dislocation of ‘social fabric’ and a threat to the ‘citizens’ rights’—calls for ‘comity, calm and commonsense’.^109^ The critic will then argue: if we exclude parliamentary privilege in the context of judicial review, we will topple the political–judicial balance, which was underpinned by a judicial respect for parliamentary privilege. Disastrous consequences will then ensue.

Three points can be made in reply. First, there is some tension between the second argument (self-defence mechanisms) and this argument. The second argument is based on the premise that parliamentary privilege—by offering *friction* between Parliament and the courts—is a justified self-defence mechanism. Its point is precisely to introduce tension into what is otherwise a relationship of comity. Yet this argument condemns any move that may raise the tension between Parliament and the courts. Therefore, a critic relying on both these arguments will have to show how it is consistent for him to push for comity *and* friction concurrently.

Second, the argument is speculative. It assumes that when courts erode parliamentary privilege, Parliament will react strongly. It further assumes that when Parliament reacts strongly, the consequences will be disastrous. Both assumptions are contestable. The first assumption is questionable because, as Lord Phillips noted in *Chaytor*, ‘extensive inroads have been made into areas that previously fell within the exclusive cognisance of Parliament’.^110^ Parliament has itself been happy to surrender grounds that previously belonged to parliamentary privilege. Indeed, in *Miller (No 2)*, the court has unequivocally affirmed the proposition that ‘it is for the court...to determine the existence and scope of [p]arliamentary privilege’.^111^ But we did not see that the judicial–political relationship has been toppled as a result. The second assumption is also contestable. Even *if* Parliament is unsatisfied with the erosion of parliamentary privilege, it can act swiftly and easily: it can enact legislation to reinstate the privilege. And that will be the end of the discussion: the judiciary will have little means to retaliate. It is quite unlikely that there will be a humungous political row between the two branches. And even granting this possibility, it is unlikely to place the protection of citizens’ rights at risk: Parliament and courts will still perform their roles in protecting human rights. It is quite unlikely that, by reason of a dispute about parliamentary privilege, the courts will stop enforcing human rights standards and Parliament will begin legislating in utter disregard of human rights.

Third, it is doubtful the deterioration of the judicial–political relationship is a matter which the courts will take into account. To say so would be tantamount to saying that the courts must, apart from propounding and applying principles of law, also consider the political consequences of potential retaliation by Parliament. Insofar as this argument is made to justify a complete defence to judicial review, this will be an astounding proposition. The courts have long made decisions that may have drastic political consequences and can easily attract political retaliation. Many landmark decisions—such as the *Case of Proclamations*,^112^*Entick*,^113^*Somerset*^114^ and the two *Miller* judgments^115^—prove that the courts will faithfully apply principles of law without fear for their political repercussions. As the majority wrote in *Miller (No 1)*, political issues are ‘matters for ministers and Parliament to resolve. They are not issues which are appropriate for resolution by judges.’ The court’s duty is singular: to ‘decide issues of law which are brought before them’.^116^

### D. Efficiency of Parliamentary Proceedings

The critic may further resort to the argument of inefficiency. It posits that removing parliamentary privilege as a defence of judicial review will hinder an efficient legislative process in Parliament. The argument has two dimensions. The first dimension is one of principle. It predicates upon the efficiency model: that since judicial review of the internal affairs of Parliament *hinders* efficient parliamentary function, the efficiency model requires the preservation of parliamentary privilege. The second dimension is one of practical consequences. It argues that, quite regardless of principle, removing parliamentary privilege will permit judicial challenges into the legislative process. This prospect will greatly inconvenience the discharge of functions of Parliament: and parliamentary privilege should be preserved to avert this danger.^117^ For instance, in *Leung*, the court justified the privilege by saying:

The important responsibilities of [the legislature], notably its law-making function, require … that it should be left to manage and resolve its own internal affairs, free from intervention by the courts and *from the possible disruption, delays and uncertainties which could result from such intervention. Freedom from these problems is both desirable and necessary in the interest of the orderly, efficient and fair disposition of [the legislature’s] business*.^118^

There are three responses to this argument (in both its dimensions). The first response is that this argument assumes that judicial review will *greatly* inconvenience Parliament. This assumption is necessary because no one would seriously suggest that a *minor* inconvenience for Parliament would justify a wholesale defence against judicial review, pursuant to either the efficiency model or the practical consequences. To make this assumption, however, the critic will have to establish that an applicant can readily apply for judicial review of the internal affairs of Parliament. There are at least three difficulties that such an applicant will have to overcome.

The first difficulty is that courts will reject premature challenges. ‘[I]n general’, the court will not ‘restrain the legislature from making unconstitutional laws’.^119^ Removing parliamentary privilege will *not* alter this rule. This is because the rule was based not only upon parliamentary privilege—it was also based upon parliamentary sovereignty,^120^ as well as the principle that the courts will not consider ‘factual matters’ until they are proven to have occurred.^121^ The latter principle applies to pre-enactment challenges because ‘it is simply unclear whether the objected provision would become law’.^122^ Neither of these two bases will be affected by removing parliamentary privilege. Thus, removing parliamentary privilege will likely only enable a challenge into the processes of Parliament, rather than a pre-enactment challenge into the *substance* of a bill (eg based on the violation of a convention right). Even if such a challenge is possible, it will not greatly inconvenience the UK Parliament. There is no higher written constitution that limits Parliament’s authority to legislate.^123^ It may, as it wishes, enact contrary to convention rights. A judicial challenge under the Human Rights Act 1998 (HRA) may not directly render the primary legislation invalid.^124^ This means even if a challenge into the substance of a bill succeeds, the court *cannot* restrain Parliament from enacting laws contrary to convention rights (and, it follows, from continuing the relevant parliamentary proceedings).

The second difficulty is standing. It is trite the degree of interests required to establish standing depends on context. When decisions ‘[affect] the public generally’, the courts will take a more liberal approach.^125^ But when it comes to a challenge of the internal procedures of Parliament, the impugned decision is unlikely to affect the public generally: it affects—as its primary victims—the MPs aggrieved by the decision, such as those in *Bradlaugh* and *McGuinness*. Therefore, given the existence of ‘an obvious challenger’ with ‘a greater and direct interest in the matter’, it will be difficult for members of the public apart from these MPs to lodge a challenge.^126^ But this does not thereby mean that MPs have a free reign to lodge judicial review. For an MP, sustaining a judicial review application which *greatly* inconvenience Parliament will likely come at the cost of political pressure towards himself and his affiliated party (let alone the accompanying financial costs). These political and financial constraints provide a strong incentive for MPs to exercise restraint before initiating a judicial challenge.

The third difficulty is the requirement of reasonable arguability. The court will refuse leave, unless ‘there is an arguable ground for judicial review having a realistic prospect of success’.^127^ So MPs cannot simply resort to judicial challenge whenever they are unsatisfied, or wish to delay parliamentary proceedings; they must have a sufficiently meritorious cause of action. This will not be an easy task to establish. Let us take, for instance, a challenge based on illegality. The MP will have to show that the Speaker has flouted or misinterpreted the rules of parliamentary procedure. This is possible, but unlikely—given how familiar the Speaker is with such rules.

As a result of these three difficulties, judicial challenges that may greatly inconvenience Parliament are limited. Most (if not all) of them will have to be based on a decision on the procedures (as opposed to the substance of the bill), lodged by an MP (who is subject to the inherent political and financial constraints) and be reasonably arguable. This casts doubt on the inefficiency argument in both its dimensions.

Then we come to the second response: the argument is predicated on the premise that if Parliament will be greatly inconvenienced, the court should refrain from judicial review altogether. In other words, the inefficiency justifies the non-justiciability which parliamentary privilege confers on Parliament. This premise is contestable on two grounds. If one adopts the efficiency model, it is accepted that inefficiency may justify judicial restraint. But it must be remembered that, according to the efficiency model, the courts remain the best candidate for resolving questions of law within Parliament. In other words, the efficiency model is both for *and* against parliamentary privilege. Therefore, while the efficiency model may require judicial *restraint* over internal affairs within Parliament, it may not require a wholesale doctrine of *non-justiciability*.^128^ To go so far would be to ignore the tension that exists between parliamentary privilege and the efficiency model, as explained above. In addition, the liberty model does not see inefficiency as a bane. Rather, it inevitably invites inefficiency: it is through the friction between the different branches that governmental power can be restrained. So, to succeed, the critic must prove two things: first, the efficiency model *requires* non-justiciability, not mere judicial restraint; and second, we should apply the efficiency model, rather than the liberty model, in the UK Constitution. The current proponents of this argument (eg *Leung*) have done nothing close to presenting such an elaborate argument.

The third response is the limits of the inefficiency argument. Not all judicial interventions into the internal affairs of Parliament will affect its legislative functions. This is possible (subject to the above-mentioned difficulties) if the challenge relates to a matter that arose during the debates. *Leung* is such an example. But many other challenges do not relate to debates. For instance, *McGuinness* is about the use of facilities in Parliament: a matter that does not affect Parliament’s performance of its legislative functions. Accordingly, if parliamentary privilege is justified on the basis of the inefficiency argument alone, the scope of the current privilege must be curtailed.

The common theme of these three responses is *not* to deny the logic of the inefficiency argument entirely. It is accepted that inconveniences may occur when judicial challenges are lodged, once parliamentary privilege is removed as a defence to judicial review. The suggestion here is that the extent of inconvenience—and the theoretical consequences of that inconvenience—are often overblown whenever this argument is applied. It ignores the difficulties that will have to be overcome for an inconvenience to be caused; it ignores the contestable theoretical premise this argument treads upon; and it ignores the fact that parliamentary privilege extends beyond debates, so that the inefficiency argument cannot justify the entirety of parliamentary privilege as it stands.

### E. Representative Democracy

The critic may then argue that the current thesis impinges on representative democracy. It has been said that parliamentary privilege is underpinned by the ‘functionality principle’: ie without parliamentary privilege, Parliament ‘cannot effectively perform’ its democratic role.^129^ One must, however, distinguish between two possible variations of this argument: (i) removing parliamentary privilege will render proceedings in Parliament inefficient;^130^ and (ii) removing parliamentary privilege will impinge on the ‘freedom of speech’ of its members.^131^ Both variations (i) and (ii) may potentially result in the dysfunction of Parliament. Insofar as variation (i) is relied upon, this is in essence the inefficiency argument—which has been addressed above.

Insofar as the critic relies on variation (ii), it is important to note that the current thesis relates *only* to judicial review. It is not suggested that the law of defamation and criminal law should apply to MPs: such laws would deter MPs from using ‘offensive...remarks’.^132^ But it is unclear why, for instance, a judicial insistence on fair procedures in Parliament means an MP can no longer ‘say whatever he or she thinks fit in debate’.^133^ Take the facts of *Bradlaugh*, *McGuinness* and *Leung*. Even if parliamentary privilege is removed in those cases, MPs can still debate freely and perform their democratic roles. It is also unclear how rationality review would threaten freedom of speech in Parliament. As Williams argued, rationality review is analogous to proportionality review under the HRA.^134^ The latter has long been available as a post-enactment challenge to legislation; and it cannot plausibly be suggested that the HRA is *per se* incompatible with parliamentary privilege, for violating freedom of speech in Parliament. If Williams’s analysis is correct, the same can plausibly be said for rationality review.^135^

One can go even further: judicial review may *enhance* the MPs’ democratic roles. First, it deters abuses of power within Parliament. Recall the example above—where the Green Party MPs have been evicted unfairly and irrationally. Judicial review would safeguard—not infringe—representative democracy by protecting aggrieved MPs from abuses. Second, judicial review facilitates ‘value coordination’ within Parliament. Fredman argued that we must distinguish between ‘interest bargaining’ (where Parliament makes decisions based on ‘factual power’) and ‘value coordination’ (where Parliament makes decisions in accordance with reason). The former is not conducive to democracy, because it encourages the politically powerful to simply subdue the powerless. The latter is conducive to democracy, because it encourages the politically powerful to deliberate with others: to ‘appeal to’ all other parties through reason, and be ‘willing to be persuaded by’ reason.^136^ Now take an enacted law that is challenged on the ground of irrationality. If Parliament is to defend the claim, it must justify why the ‘indicia of unreasonableness’ (eg ‘disproportionality’) did not exist before a court.^137^ Laws that were made *solely* through ‘interest bargaining’ cannot withstand the challenge. Judicial review steers legislative decision making towards ‘value coordination’—which, in turn, enhances MPs’ democratic roles.^138^

This argument is not to undermine the importance of freedom of speech in Parliament. This is historically why we had parliamentary privilege in the first place. Chafetz gave us some examples of this: ‘[i]n 1397, a bill was introduced in the House of Commons condemning the extravagant expenditures of the royal household’. Richard II retaliated by having Thomas Haxey—who introduced the bill—‘declared...a traitor and condemned...to death’.^139^ In 1566, some MPs criticised Queen Elizabeth’s ‘refusal to address Parliament’s petition on...royal succession’. The queen then summoned those MPs and ‘forbade them to discuss such matters in the future’.^140^ In 1680, the Speaker ordered for a pamphlet which defamed the Duke of York (later James II) to be printed. He was later fined in court on the ground of ‘seditious libel’.^141^ It is incidents like this which underpinned the enactment of article 9—to defend the freedom of speech of MPs.^142^ The ‘freedom of speech’ remains the principal justification of parliamentary privilege today;^143^ and its importance is illustrated by the many instances where the Speaker has intervened to assert article 9.^144^ The current thesis does not deny this. It only suggests that—despite its importance—this freedom does not require *judicial review* to be entirely precluded.

### F. Parliamentary Sovereignty

The critic may finally resort to the argument based on parliamentary sovereignty. He may argue that Parliament is sovereign, and thus its laws cannot be challenged in courts.^145^ This current thesis (he may argue) is flawed, for it contradicts parliamentary sovereignty. Two responses can be made. First, this argument assumes that judicial review necessarily contradicts parliamentary sovereignty. Parliamentary sovereignty provides that Parliament can ‘make or unmake any law whatsoever’,^146^ and that the courts cannot strike down laws made by Parliament.^147^ But this does not mean the *existence* of review should go altogether. It is entirely possible for courts to make a declaration that a certain law has been improperly or irrationally made by Parliament without striking it down. Parliament can then decide on its course of action. This is compatible with parliamentary sovereignty, since Parliament remains free to make or unmake any law whatsoever. Indeed, this is the very premise of the HRA: the courts cannot directly strike down rights-incompatible primary legislation, but they may make a declaration of incompatibility.^148^ This is deliberately designed to maintain parliamentary sovereignty under the HRA.^149^

Second, this thesis—that the positive law of Parliament is unjustified—is normative in nature, sustained by a general theory of the separation of powers. Saying this thesis contradicts the positive law of Parliament does not defeat the thesis: it simply restates its premise. To object to the current thesis, the critic must proceed *normatively—*by proving either (i) Parliament should be free from the separation of powers or (ii) in any event, the separation of powers does not preclude parliamentary privilege. In its normative dimension, the argument based on parliamentary sovereignty is relevant insofar as it suggests that proposition (i) is correct. But this argument is a non-sequitur: that Parliament is sovereign does not mean it should be *entirely* unchecked by the other branches. *Chaytor*, *Miller (No 2)* and the HRA would all contradict this. So parliamentary sovereignty, properly viewed, does not provide an obstacle to the current thesis.

## 5. Conclusion

In conclusion, three propositions concerning parliamentary privilege were made in this article. First, in the context of judicial review, parliamentary privilege is defined by the outer limits of the principle of exclusive cognisance. To this, article 9 adds nothing. Second, parliamentary privilege—insofar as it provides a defence to judicial review—is incompatible with the two prevailing models of the separation of powers. Third, six arguments that may be made in opposition to this thesis are flawed. Therefore, parliamentary privilege should no longer provide a defence to judicial review. While the positive law is clearly not in favour of this thesis, the thesis shows that the positive law is normatively unjustified.

## Footnotes

1 William Blackstone, *Commentaries on the Laws of England: Book I: Of the Rights of Persons* (OUP 2016) 109.

2
*Bradlaugh v Gossett* (1884) 12 QBD 271, 275.

3
*Pepper v Hart* [1992] UKHL 3, [1993] AC 593. See eg Johan Steyn, ‘*Pepper v Hart*; A Re-examination’ (2001) 21 OJLS 59; Stefan Vogenauer, ‘A Retreat from *Pepper v Hart*?’ (2005) 25 OJLS 629; Philip Sales, ‘*Pepper v Hart:* A Footnote to Professor Vogenauer’s Reply to Lord Steyn’ (2006) 26 OJLS 585; Aileen Kavanagh, ‘Proportionality and Parliamentary Debates: Exploring Some Forbidden Territory’ (2014) 34 OJLS 443.

4
*R (Miller) v Prime Minister* [2019] UKSC 41, [2020] AC 373.

5 See eg Paul Craig, ‘The Supreme Court, Prorogation and Constitutional Principle’ [2020] PL 248, 275–7; Martin Loughlin, ‘The UK Constitutional Council’s Ruling on Appeal from the Judgment of the Supreme Court’ (*Policy Exchange*, 15 October 2019) [7]–[9].

6
*Buchanan v Jennings* [2004] UKPC 36, [2005] 1 AC 115.

7
*Ex p Wason* (1869) LR 4 QB 573.

8
*Prebble v Television New Zealand Ltd* [1994] UKPC 4, [1995] 1 AC 321, 332; Martin Chamberlain QC and James Segan, ‘Parliamentary Privilege, Article 9 of the Bill of Rights and Admissibility: What Use Can Be Made of Parliamentary Materials in Litigation?’ (2018) 23 JR 11.

9 Bill of Rights 1689, art 9.

10
*R v Chaytor* [2010] UKSC 52, [2011] 1 AC 684 [63].

11 Chamberlain and Segan (n 8) [2].

12
*Chaytor* (n 10) [61], [67]; Philip A Joseph, ‘Parliament’s Attenuated Privilege of Freedom of Speech’ (2010) 126 LQR 568, 574–5.

13
*Miller* (n 4) [66].

14
*Prebble* (n 8) 332.

15 Richard Ekins, ‘Parliamentary Sovereignty and the Politics of Prorogation’ (*Policy Exchange*, 16 September 2019) 21.

16
*Chaytor* (n 10) [102].

17 ibid 104.

18 Yvonne Tew, ‘No Longer a Privileged Few: Expense Claims, Prosecution and Parliamentary Privilege’ (2011) 70 CLJ 282, 283–4; Sir John Saunders, ‘Parliamentary Privilege and the Criminal Law’ [2017] Crim LR 521, 529–30.

19
*Chaytor* (n 10) [128]–[129].

20 See eg Joseph (n 12).

21
*Chaytor* (n 10) [28].

22 ibid [47].

23 ibid [61].

24 ibid [62].

25 ibid [63].

26 Joint Committee on Parliamentary Privilege, *Parliamentary Privilege—First Report* (HL 1998–99, 43-I) [241], cited in *Chaytor* (n 10) [72] (emphasis added).

27 Joint Committee on Parliamentary Privilege (n 26) [247], cited in *Chaytor* (n 10) [73].

28
*Miller* (n 4) [66], [68]–[69].

29
*Bradlaugh* (n 2) 284.

30 ibid 275.

31 ibid.

32 ibid 278.

33
*Re McGuinness’s Application* [1997] NIQB 2.

34 ibid.

35
*Prebble* (n 8) 332; *Chaytor* (n 10) [13].

36
*R v Parliamentary Commissioner for Standards, ex p Al Fayed* [1998] 1 WLR 669, 671.

37
*Miller* (n 4) [66], [68].

38 ibid [66].

39
*Chaytor* (n 10) [30].

40 ibid [76].

41 ibid [76]–[77].

42 ibid [61].

43 ibid [13].

44
*Leung Kwok Hung v President of the Legislative Council (No 1)* [2014] HKCFA 74, (2014) 17 HKCFAR 689.

45 ibid [28].

46 ibid [46].

47 This is subject to one difference which is not material here. The difference is that, unlike the principle of exclusive cognisance, the principle in Hong Kong law is subject to the requirements of the written constitution: see *Bahamas District of the Methodist Church in the Caribbean and the Americas v The Hon Vernon J Symonette MP (Bahamas)* [2000] UKPC 31 [27], [29]–[30]; *Chief Executive of the Hong Kong Special Administrative Region v The President of the Legislative Council* [2016] HKCA 576, [2017] 1 HKLRD 460 [24]–[25].

48
*Secretary for Justice v Leung Kwok Hung* [2020] HKCA 424 [36].

49 NW Barber, *The Principles of Constitutionalism* (OUP 2018) 76–7.

50 ibid 77.

51 ibid.

52 ibid 78.

53 Eric Barendt, ‘Separation of Powers and Constitutional Government’ [1995] PL 599, 614.

54 Barber (n 49) 66.

55 ibid 67.

56 Barendt (n 53) 615.

57 ibid 606.

58 Laurence Claus, ‘Montesquieu’s Mistakes and the True Meaning of Separation’ (2005) 25 OJLS 419, 434–5.

59 ibid 419, 425; NW Barber, ‘Self-defence for Institutions’ (2013) 72 CLJ 558, 565; Barber, *The Principles of Constitutionalism* (n 49) 52.

60 Barber, *The Principles of Constitutionalism* (n 49) 52.

61 Barendt (n 53) 607.

62 See eg *Anisminic v Foreign Compensation Commission* [1968] UKHL 6, [1969] 2 AC 147; *R v Medical Appeal Tribunal, ex p Gilmore* [1957] 1 QB 574, 583.

63
*R (Cart) v The Upper Tribunal* [2009] EWHC 3052 (Admin), [2010] 2 WLR 1012 [38]. This has already been said to be a requirement of the separation of powers: see TRS Allan, *Law, Liberty, and Justice: The Legal Foundations of British Constitutionalism* (Clarendon Press 1994) 65–8; TRS Allan, ‘Accountability to Law’ in Nicholas Bamforth and Peter Leyland (eds), *Accountability in the Contemporary Constitution* (OUP 2013) 97–8.

64
*R v Shoreditch Assessment Committee, ex p Morgan* [1910] 2 KB 859, 880. See also the work by Allan cited in n 63.

65 Barendt (n 53) 614.

66 Barber, ‘Self-defence for Institutions’ (n 59) 66–7.

67 Andrew Geddis, ‘Parliamentary Privilege: quis custodiet ipsos custodes?’ [2005] PL 696, 703; Graeme Watt, ‘Proceedings in Parliament’ [2019] PL 469, 470–1; *Chaytor* (n 10) [14]–[15].

68 Murdoch Thomson, ‘Parliamentary Privilege in *R v White (Lord Hanningfield)* 2016: All Equal Before the Law?’ (2017) 29 Denning Law Journal 131, 136–7.

69
*Chaytor* (n 10) [78].

70 ibid [74].

71 Barber, ‘Self-defence for Institutions’ (n 59) 566.

72 ibid 568; Aileen Kavanagh, ‘The Constitutional Separation of Powers’ in David Dyzenhaus and Malcolm Thorburn (eds), *Philosophical Foundations of Constitutional Law* (OUP 2016) 231.

73 Barber, *The Principles of Constitutionalism* (n 49) 57–8.

74 ibid 65–6.

75 ibid 63–4.

76 ibid 58–9.

77 Kavanagh, ‘The Constitutional Separation of Powers’ (n 72) 235.

78 Above n 44.

79
*McGuinness v The United Kingdom* App no 39511/98 (Third Section, 8 June 1999).

80 David Natzler and others, *Erskine May: Parliamentary Practice* (25th edn, LexisNexis Butterworths 2019).

81 Barber, *The Principles of Constitutionalism* (n 49) 63-64. See also Danny Gittings, ‘Separation of Powers in Hong Kong: Inching Towards a More Flexible Judicial Interpretation’ (2019) 49 HKLJ 187, 206.

82
*Miller* (n 4) [31].

83
*R v Lord President of the Privy Council, ex p Page* [1992] UKHL 12, [1993] AC 682.

84
*R v Secretary of State for the Home Department, ex p Doody* [1993] UKHL 8, [1994] 1 AC 531.

85
*Associated Provincial Picture Houses Ltd v Wednesbury Corporation* [1947] EWCA Civ 1, [1948] 1 KB 223.

86 A further objection may be that discarding parliamentary privilege encourages judicial intervention into parliamentary proceedings. While judges may be more suited to determine questions of law, it burdens the efficiency of parliamentary proceedings. This undermines the very aim of the efficiency account—to promote efficient state action. Accordingly, the efficiency account would in fact mandate parliamentary privilege. Due to its complexity, this argument will be addressed at length below.

87 MJC Vile, *Constitutionalism and the Separation of Powers* (2nd edn, Liberty Fund 1998) 13.

88 ibid 13; Kavanagh, ‘The Constitutional Separation of Powers’ (n 72) 224.

89
*Bahamas* (n 47) [27] (emphasis added).

90
*Leung* (n 44) [28] (emphasis added).

91 ibid (emphasis added).

92 Claus (n 58) 443; Kavanagh, ‘The Constitutional Separation of Powers’ (n 72) 225–6; Dimitros Kyritsis, *Where Our Protection Lies: Separation of Powers and Constitutional Review* (OUP 2017) 37; Gittings (n 81) 189.

93 Kavanagh, ‘The Constitutional Separation of Powers’ (n 72) 225.

94 ibid.

95 Barber, ‘Self-defence for Institutions’ (n 59) 560.

96 ibid.

97 ibid 564, 568, 570.

98 ibid 570–1.

99 ibid 571, 576.

100 ibid 572–573, 579; Barber, *The Principles of Constitutionalism* (n 49) 72–73.

101 Barber, *The Principles of Constitutionalism* (n 49) 81–2; see also Barber, ‘Self-defence for Institutions’ (n 59) 572–3, 575.

102 Barber, ‘Self-defence for Institutions’ (n 59) 573; Philip Sales, ‘Partnership and Challenge: The Courts’ Role in Managing the Integration of Rights and Democracy’ [2016] PL 456, 466–9.

103
*Miller* (n 4) [31].

104 Barber, *The Principles of Constitutionalism* (n 49) 81–2; see also Barber, ‘Self-defence for Institutions’ (n 59) 572–3, 575, 579.

105 Barber, ‘Self-defence for Institutions’ (n 59) 573.

106 Paul Craig, ‘Proportionality, Rationality and Review’ [2010] NZLR 265, 296–7; Paul Craig, ‘The Nature of Reasonableness Review’ (2013) 66 CLP 131, 161.

107 Joseph (n 12) 570; Evan Fox-Decent, ‘Parliamentary Privilege, Rule of Law and the Charter after the *Vaid* Case’ (2007) (Autumn) Canadian Parliamentary Review 27, 28; Conor Crummey and Eugenio Velasco Ibarra, ‘Statutory Conventions: Conceptual Confusion or Sound Constitutional Development?’ [2018] PL 613, 623.

108
*British Railways Board v Pickin* [1974] UKHL 1, [1974] AC 765, 799; see also *Bradlaugh* (n 2) 286–7.

109 Joseph (n 12) 570; Lord Burnett, ‘Parliamentary Privilege—Liberty and Due Limitation’ [2019] JR 107 [22].

110
*Chaytor* (n 10) [78].

111
*Miller* (n 4) [66]; see also ibid [14]–[15].

112 (1611) 12 Co Rep 74.

113
*Entick v Carrington* (1765) 19 St Tr 1030.

114
*Somerset v Stewart* (1772) 98 ER 499.

115
*R (Miller) v Secretary of State for Exiting the European Union* [2017] UKSC 5, [2018] AC 61; *Miller* (n 4).

116
*R (Miller)* (n 115) [3].

117 Serge Joyal, ‘The *Vaid* Case and the Protection of Parliamentary Employees Against Human Rights Discrimination’ (2005) (Winter) Canadian Parliamentary Review 2, 2; Warren J Newman, ‘Parliamentary Privilege, the Canadian Constitution and the Courts’ (2007) 39 Ottawa L Rev 573, 592.

118
*Leung* (n 44) [30] (emphasis added); see also *Canada (House of Commons) v Vaid* [2005] 1 SCR 667 [20].

119
*Bahamas* (n 47) [25].

120 ibid [25]–[26].

121
*R (Yalland) v Secretary of State for Exiting the European Union* [2017] EWHC 630 (Admin) [23].

122
*Leung Lai Kwok Yvonne v Chief Secretary for Administration* [2015] HKCFI 929 [40].

123
*R (Miller)* (n 115) [43]; Craig, ‘The Supreme Court’ (n 5) 254.

124 See eg *RR v Secretary of State for Work and Pensions* [2019] UKSC 52, [2019] 1 WLR 6430 [16], [28]–[29].

125
*AXA General Insurance Ltd v HM Advocate* [2011] UKSC 46, [2012] 1 AC 868 [170].

126
*Li Fung Kei v The Council of the University of Hong Kong* [2018] 4 HKC 231 [49(3)].

127
*Sharma v Deputy Director of Public Prosecutions (Trinidad and Tobago)* [2006] UKPC 57, [2007] 1 WLR 780 [14(4)].

128 For the difference between the two, see Aileen Kavanagh, ‘Defending Deference in Public Law and Constitutional Theory’ (2010) 126 LQR 222, 241–2.

129 Richard Gordon QC and Sir Malcolm Jack KCB, PhD, FSA, ‘Parliamentary Privilege: Evolution or Codification?’ (*The Constitutional Society*, 2013) [11], [14]–[15]; Joint Committee on Parliamentary Privilege (n 26) [3]–[4], [23]. That this may underpin the great weight that courts place on parliamentary privilege, see *R (HS2 Action Alliance Ltd) v Secretary of State for Transport* [2014] UKSC 3, [2014] 1 WLR 324 [79].

130 Joint Committee on Parliamentary Privilege (n 26) [13], [230]–[240].

131 Gordon and Jack (n 129) [29]; Joint Committee on Parliamentary Privilege (n 26) [12], [36].

132 Gordon and Jack (n 129) [28].

133 ibid [29].

134 Rebecca Williams, ‘Structuring Substantive Review’ [2017] PL 99.

135 This is especially since, as explained earlier, a pre-enactment challenge against Parliament into the substance of a bill will likely remain barred. So rationality review, like proportionality review under the HRA, will only be available post-enactment.

136 Sandra Fredman, *Human Rights Transformed: Positive Rights and Positive Duties* (OUP 2008) 35–6; Sandra Fredman, *Comparative Human Rights Law* (OUP 2018) 83–4. This is why it was said that ‘[t]he legitimacy of democratic decisions...emerge[s] from a deliberative process open to all’—including one where the minority may challenge a majority decision: see Melissa Schwartzberg, *Democracy and Legal Change* (CUP 2007) 203–4. This minority challenge would not be possible if only ‘interest bargaining’ is adopted by Parliament, for then the majority would simply ignore the minority.

137 Paul Daly, ‘*Wednesbury*’s Reason and Structure’ [2011] PL 238, 242–3.

138 Fredman, *Human Rights Transformed* (n 136) 105; Fredman, *Comparative Human Rights Law* (n 136) 91–3.

139 Josh Chafetz, *Democracy’s Privileged Few: Legislative Privilege and Democratic Norms in the British and American Constitutions* (Yale UP 2007) 69.

140 ibid 70–1.

141 ibid 74.

142 ibid 74–5. This is also the reason why it makes sense for art 9 to exist, although it adds nothing to the principle of exclusive cognisance in the context of judicial review: the former goes beyond judicial review. For instance, it safeguards the freedom of speech—through precluding the law of defamation in parliamentary proceedings.

143 Gordon and Jack (n 129) [29]; *Office of Government v Information Commissioner* [2008] EWHC 737 (Admin), [2010] QB 98 [46]; *Kimathi v Foreign and Commonwealth Office* [2017] EWHC 3379 (QB), [2018] 4 WLR 48 [5]–[7].

144 See eg *R (Federation of Tour Operators) v HM Treasury* [2007] EWHC 2062 (Admin); *R (Wheeler) v Office of the Prime Minister* [2008] EWHC 1409 (Admin); *R (Bradley) v Secretary of State for Work and Pensions* [2009] QB 114; *Office of Government* (n 143); *Kimathi* (n 143); *R (Heathrow Hub Ltd) v The Secretary of State for Transport* [2019] EWHC 1069 (Admin); *R (Heathrow Hub Ltd) v Secretary of State for Transport* [2020] EWCA Civ 213.

145
*Pickin* (n 108) 787–90; *AXA* (n 125) [48]–[49].

146
*R (Miller)* (n 115) [43]; Farrah Ahmed and Adam Perry, ‘The Quasi-entrenchment of Constitutional Statutes’ (2014) 73 CLJ 514, 534; Craig, ‘The Supreme Court’ (n 5) 254.

147
*R (Miller)* (n 115) [43]; *Pickin* (n 108) 787–90; *AXA* (n 125) [48]–[49].

148 Jeremy Waldron, ‘The Core of the Case Against Judicial Review’ (2006) 115 YLJ 1346, 1355. This form of judicial review has been said to constitute a dialogue between Parliament and the courts: see Alison L Young, ‘Is Dialogue Working under the Human Rights Act 1998?’ [2011] PL 773. The same, it is suggested, can be said of the kind of judicial review envisaged here.

149 Nicholas Bamforth, ‘Parliamentary Sovereignty and the Human Rights Act 1998’ [1998] PL 572; Richard Ekins, ‘Legislative Freedom in the United Kingdom’ (2017) 133 LQR 582, 594.

